# Assessing Data Quality in Heterogeneous Health Care Integration: Simulation Study of the AIDAVA Framework

**DOI:** 10.2196/75275

**Published:** 2025-11-12

**Authors:** Jens Declerck, Ömer Durukan Kılıç, Ensar Emir Erol, Shervin Mehryar, Dipak Kalra, Isabelle de Zegher, Remzi Celebi

**Affiliations:** 1 The European Institute for Innovation Through Health Data Oosterzele Belgium; 2 Department of Public Health and Primary Care Unit of Medical Informatics and Statistics Ghent University Ghent Belgium; 3 Department of Advanced Computing Sciences Institute of Data Science Maastricht University Maastricht The Netherlands; 4 B!lboa Tervuren Belgium

**Keywords:** data quality, knowledge graph, ontology, health data, data quality dimensions, data quality assessment, secondary use, data quality framework, fit for purpose

## Abstract

**Background:**

Integrated health data are foundational for secondary use, research, and policymaking. However, data quality issues—such as missing values and inconsistencies—are common due to the heterogeneity of health data sources. Existing frameworks often use static, 1-time assessments, which limit their ability to address quality issues across evolving data pipelines.

**Objective:**

This study evaluates the AIDAVA (artificial intelligence–powered data curation and validation) data quality framework, which introduces dynamic, life cycle–based validation of health data using knowledge graph technologies and SHACL (Shapes Constraint Language)–based rules. The framework is assessed for its ability to detect and manage data quality issues—specifically, completeness and consistency—during integration.

**Methods:**

Using the MIMIC-III (Medical Information Mart for Intensive Care-III) dataset, we simulated real-world data quality challenges by introducing structured noise, including missing values and logical inconsistencies. The data was transformed into source knowledge graphs and integrated into a unified personal health knowledge graph. SHACL validation rules were applied iteratively during the integration process, and data quality was assessed under varying noise levels and integration orders.

**Results:**

The AIDAVA framework effectively detected completeness and consistency issues across all scenarios. Completeness was shown to influence the interpretability of consistency scores, and domain-specific attributes (eg, diagnoses and procedures) were more sensitive to integration order and data gaps.

**Conclusions:**

AIDAVA supports dynamic, rule-based validation throughout the data life cycle. By addressing both dimension-specific vulnerabilities and cross-dimensional effects, it lays the groundwork for scalable, high-quality health data integration. Future work should explore deployment in live clinical settings and expand to additional quality dimensions.

## Introduction

### Background

The integration of high-quality, complete, and interoperable patient health records is essential to modern health care and medical research [1-4]. Accurate and well-structured data enhance research reproducibility [5-7], which in turn drives more effective clinical decision-making and improved patient outcomes. However, as health data is collected across diverse and heterogeneous sources [8,9], its quality can be compromised by fragmentation [10,11], variability [12,13], and incomplete information [14-16]. These challenges compromise data usability and hinder the development of unified, clinically meaningful datasets suitable for both primary and secondary uses [17,18].

Existing efforts on health data quality often focus on defining standardized quality dimensions and organizing these into structured frameworks [19-23]. However, many current approaches rely on static, 1-time evaluations that do not reflect the dynamic and iterative nature of the entire data life cycle [24,25]. This limits their effectiveness in identifying evolving quality issues that emerge across stages of data transformation—such as extraction [26,27], harmonization [28,29], or final validation [21]—as they do not provide continuous, iterative assessment.

To address this gap, the AIDAVA (artificial intelligence–powered data curation and validation) project [30], launched in 2022 as part of a Horizon Europe initiative, proposes a dynamic data quality framework that enables continuous assessment throughout the data life cycle. At its core is the personal health knowledge graph (PHKG), a patient-centered, interoperable data model built using knowledge graph (KG) technologies and validated with SHACL (Shapes Constraint Language)–based rules. This approach allows for the assessment of data quality constraints across multiple integration stages. For instance, if a patient record includes a diagnosis of prostate cancer but the patient is listed as a female, or if a discharge date appears earlier than the admission date, AIDAVA’s rule-based validation will automatically detect and flag these inconsistencies during the integration process.

This paper also demonstrates how completeness directly influences the interpretability of consistency scores.

This paper evaluates the AIDAVA framework’s effectiveness in detecting and improving data quality issues, with a particular focus on completeness and consistency. The framework’s ability to validate data dynamically across stages of integration is essential for supporting artificial intelligence–driven, automated curation workflows, a central goal of the AIDAVA project. As health care systems increasingly rely on semantic technologies and automation to manage large-scale, heterogeneous data, life cycle–based quality monitoring becomes a requirement. By situating this work within that broader vision, we aim to demonstrate how dynamic rule-based validation can enhance the reliability and scalability of next-generation health data integration pipelines. It also investigates how these dimensions evolve across the data transformation pipeline, highlighting their interdependencies and implications for integrated health data (Textbox 1).

**Main contributions of this paper.**

- Introduces the AIDAVA (artificial intelligence–powered data curation and validation) framework for dynamic health data quality validation using SHACL (Shapes Constraint Language) and knowledge graphs.
- Defines and applies completeness and consistency rules across the integration pipeline.
- Simulates realistic data quality issues using controlled noise in the MIMIC-III (Medical Information Mart for Intensive Care-III) dataset.
- Evaluates SHACL validation across different integration sequences and noise levels.

### Related Work

Ensuring the quality of integrated health care data, particularly for the secondary use of electronic health records, has been the subject of extensive research [31]. Prior efforts generally focus on defining and measuring data quality along dimensions such as completeness, consistency, conformance, and plausibility [20-24]. These dimensions serve as the foundation for several frameworks and tools developed to detect anomalies and enforce standards across clinical datasets [15,22,32].

Framework-based approaches have sought to structure and formalize the assessment of health data quality. Notably, Kahn et al [21] introduced a widely adopted framework that groups quality concerns into 3 core dimensions—completeness, conformance, and plausibility—further divided into verification and validation contexts. This framework laid the groundwork for systematic quality checks but remains limited to static evaluations that do not adapt to the changing nature of data pipelines.

Dimension-specific studies have also provided deeper insights. Issa et al [33] conducted a comprehensive review of completeness in KGs, identifying 7 distinct subtypes, including 3 previously unclassified forms. Their findings emphasized the interplay between completeness and other quality dimensions such as consistency and correctness—further highlighting the need for more context-aware, dynamic quality assessments.

Tool-based solutions, such as Achilles Heel [34], offer practical mechanisms for identifying data quality issues. As part of the Observational Health Data Sciences and Informatics (OHDSI) ecosystem, Achilles Heel applies 70 predefined validation rules to detect anomalies in large-scale clinical datasets. While effective in identifying static inconsistencies, these tools typically evaluate data quality at a single point in time and cannot accommodate the iterative transformations that occur during integration workflows.

In contrast to these prior approaches, the AIDAVA framework introduces a life cycle–based, dynamic model of data quality validation. By embedding SHACL-based validation rules directly into a KG pipeline, AIDAVA allows for continuous monitoring and enforcement of constraints during data ingestion, transformation, and integration. It extends existing methodologies by addressing data quality not as a 1-time task, but as an ongoing process—ensuring that completeness and consistency are maintained across evolving data structures.

### AIDAVA Data Quality Framework

**Figure 1:**
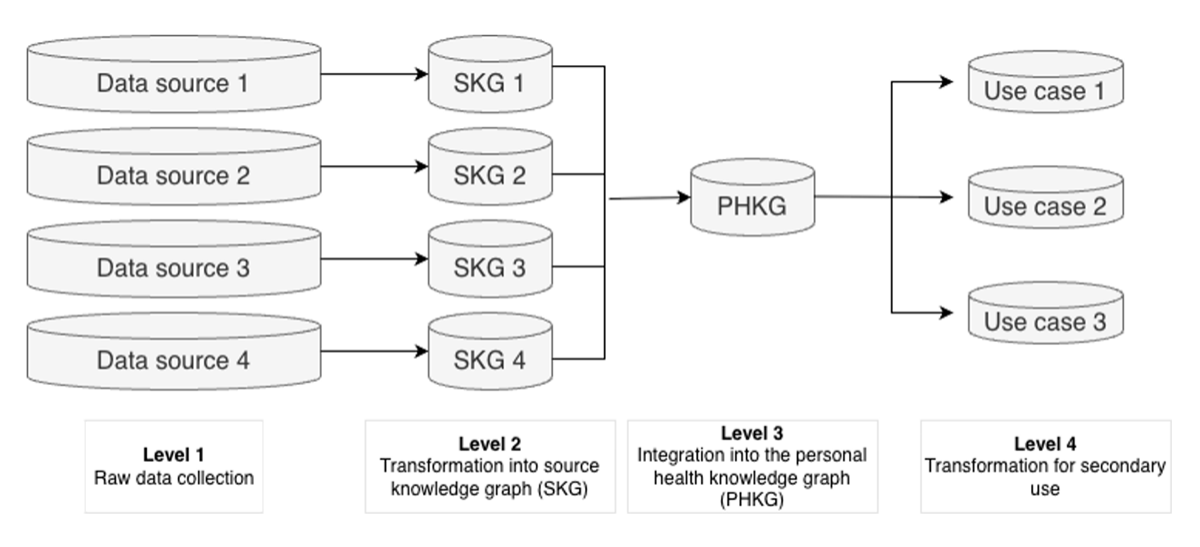
The 4 levels of the AIDAVA data quality framework. AIDAVA: artificial intelligence–powered data curation and validation; PHKG: personal health knowledge graph; SKG: source knowledge graph.

The AIDAVA data quality framework is designed to ensure semantic and structural standardization across the entire health data life cycle, addressing the complexities of integrating heterogeneous health data sources. The framework operates across 4 levels, each targeting specific points in the health data integration process. These 4 levels of the framework are illustrated in Figure 1.

### Level 1: Raw Data Collection

At the initial level, the framework relies on data providers to provide data that meets baseline standards. Given the variability of data sources, direct transformations or validations at this stage are limited. Instead, the framework ensures compliance with transfer specifications, verifying that incoming data adheres to structural and format requirements before progressing to the next level.

### Level 2: Transformation Into Source Knowledge Graphs

In this stage, raw data are transformed into source knowledge graphs (SKG) by executing a data curation tool from the library of tools available in AIDAVA and delivering data sources in a KG format aligned with the AIDAVA Reference Ontology. The AIDAVA Reference Ontology plays a dual role in enabling semantic interoperability and systematic quality evaluation. It provides a formal semantic layer that aligns PHKGs with standards such as Health Level Seven International Fast Healthcare Interoperability Resources (FHIR), SNOMED CT (Systematized Nomenclature of Medicine–Clinical Terms), and Clinical Data Interchange Standards Consortium. This process ensures semantic and structural standardization of each data source, which is critical for interoperability. Standardizing the data at this level minimizes inconsistencies and enables proper integration with multiple sources.

### Level 3: Integration Into the PHKG

In this phase, multiple SKGs are integrated into a unified PHKG, creating a longitudinal representation of an individual’s health history. This step consolidates fragmented patient data sources while maintaining consistency and logical coherence in the integrated patient record. The integration process ensures that patient data is well-structured, complete, and free of inconsistent and redundant information to support clinical decision-making and research applications. For example, during PHKG integration, a birth year recorded as 1875, implying an implausible age of over 140 years, would be flagged by age-consistency rules. This type of anomaly is automatically detected through SHACL-based validation during the integration process.

### Level 4: Transformation for Secondary Use

In the final stage, the PHKG is transformed into formats tailored for secondary use. This step adapts integrated data to align with specific output formats, enabling accurate analysis (eg, Breast Cancer registry in Observational Medical Outcomes Partnership [OMOP] compliant format), improved patient care (eg, patient International Patient Summary in FHIR format), and effective reuse across clinical and research settings.

This study focuses on levels 2 and 3, as they are the earliest feasible and scalable stages for enforcing semantic standardization and integrating fragmented, heterogeneous data sources. Performing this at level 1 is not practical due to diverse formats and limited control over data provider systems. Level 4 is only partially addressed, as downstream transformation is performed, but full data quality assessment from the perspective of a specific use case lies outside the scope of this paper.

### Data Quality Dimensions and Categories

To evaluate data quality, the AIDAVA framework adopts a structured approach based on dimensions and categories. Dimensions provide a high-level perspective on data quality, grouping related categories to help identify systemic issues. Recent research underscores the increasing need to evaluate diverse data quality dimensions, especially when repurposing health data for secondary use [19]. While multiple dimensions contribute to ensuring high-quality health data, the AIDAVA framework currently focuses on 2 data quality dimensions: completeness and consistency. Completeness ensures that all necessary data elements are present, preventing critical information gaps. Consistency verifies that data adheres to defined constraints and logical relationships, such as ensuring that diagnoses and procedures align with a patient’s demographic details. Within these dimensions, categories provide a more granular level of assessment, as outlined in Table 1.

The decision to focus on completeness and consistency is driven by their widespread use in data quality research [35-37] and their suitability for automated assessment [23]. Unlike other dimensions, which often require subjective interpretation or manual validation, completeness and consistency can be systematically measured using predefined rules and automated validation techniques. By prioritizing completeness and consistency, the AIDAVA framework establishes a scalable approach to data quality assessment. These dimensions not only support automated validation but also provide a foundation for expanding the framework to address more complex data quality dimensions.

**Table 1:** Overview of data quality dimensions and categories in the AIDAVA^a^ framework.

Dimension and category		Description	Example
**Completeness**			
	Essential variable completeness	Identifies when critical data elements are missing	Missing patient date of birth
	Conditional variable completeness	Detects cases where missing variables prevent consistency checks	Diagnosis present, but no recorded gender
**Consistency**			
	Data type for property	Ensures values conform to expected data types	Text found in a numeric age field
	Time sequence consistency	Identifies implausible event sequences	Discharge date before admission date
	Diagnosis for gender consistency	Detects diagnoses incompatible with a patient’s gender	Prostate cancer assigned to a female patient
	Diagnosis for age consistency	Flags diagnoses that are incompatible with a patient’s age	Lung cancer diagnosed in an infant
	Procedure for gender consistency	Detects gender-incompatible procedures	Hysterectomy assigned to a male patient
	Procedure for age consistency	Flags procedures that are inappropriate for the patient’s age	Radiotherapy procedure in a toddler

^a^AIDAVA: artificial intelligence–powered data curation and validation.

### Data Quality Instruments

The AIDAVA framework relies on a robust instrument for scalable and automated data quality assessment: the SHACL. As a World Wide Web Consortium standard, SHACL defines and enforces semantic and structural constraints on Resource Description Framework (RDF) KGs, enabling rule-based validation to detect inconsistencies, missing elements, and structural misalignments.

Within the AIDAVA framework, SHACL validation rules are categorized into 2 types, as outlined in Table 2: ontology-based and domain-specific rules. Each category includes several validation rules, technically known as SHACL shapes—an SHACL term referring to rule templates that define how data should conform to expected structures or values. The number of rules differs between categories due to the nature of the information being encoded. For instance, completeness checks such as “essential variable” rely on the fully connected structure of the admission node to validate the presence of key attributes (eg, admission date and discharge status) across sources. In contrast, rules such as “diagnosis for gender” require a distinct SHACL shape for each valid gender related pairing (eg, “prostate cancer – male” and “ovarian cancer – female”), increasing the number of shapes. Table 2 shows the number of SHACL shapes associated with each rule category, reflecting the complexity and granularity of the validations.

To enable meaningful evaluation of data quality across different rule categories, we use normalized data quality scores rather than raw violation counts. This is necessary because the number of rules (SHACL shapes) and corresponding checked nodes varies significantly by category (Table 2). Violation counts alone would disproportionately reflect rule volume rather than actual quality trends. We calculate a category-specific quality score using the formula:

*Quality score* = 1 – *e*

Where *e* represents the error rate, calculated as the violation count divided by the total number of checked nodes. This provides a relative measure of how well the data conforms to the specified rules within each category.

**Table 2:** Categories and counts of SHACL^a^ validation rules in the AIDAVA^b^ framework.

Type and category		SHACL shape count
**Ontology-based checks**		
	Essential variable completeness (eg, admission date must be present)	14
	Conditional variable completeness (eg, discharge status cannot be validated without discharge time)	1
	Data type for property (eg, age must be a number, not text)	6
**Medical and common-sense checks**		
	Time sequence consistency (eg, admission after discharge flagged as invalid)	6
	Diagnosis for gender consistency (eg, female patient assigned prostate cancer code)	5208
	Diagnosis for age consistency (eg, infant patient assigned prostate cancer code)	130
	Procedure for gender consistency (eg, male patient assigned hysterectomy code)	640
	Procedure for age consistency (eg, infant patient assigned colonoscopy procedure)	79

^a^SHACL: Shapes Constraint Language.

Ontology-based rules in the AIDAVA framework are derived from the AIDAVA reference ontology [38], which builds on established standards such as Health Level Seven International FHIR, SNOMED (Systematized Nomenclature of Medicine), and LOINC (Logical Observation Identifiers Names and Codes) to ensure interoperability and support automated curation. These rules ensure conformance with predefined semantic standards, such as verifying data types, relationships, and the presence of mandatory variables. Domain-specific rules are informed by health care–specific knowledge. These rules address real-world data quality challenges, including validating gender-appropriate procedures and ensuring consistency in clinical attributes. The definition and validation of domain-specific rules require expert consensus. However, data quality research has established a variety of validated domain-specific rules over time [39]. The AIDAVA framework leverages these existing, literature-validated rules, allowing it to build upon proven methodologies while avoiding redundancy, ensuring alignment with best practices in health data quality management.

**Figure 2:**
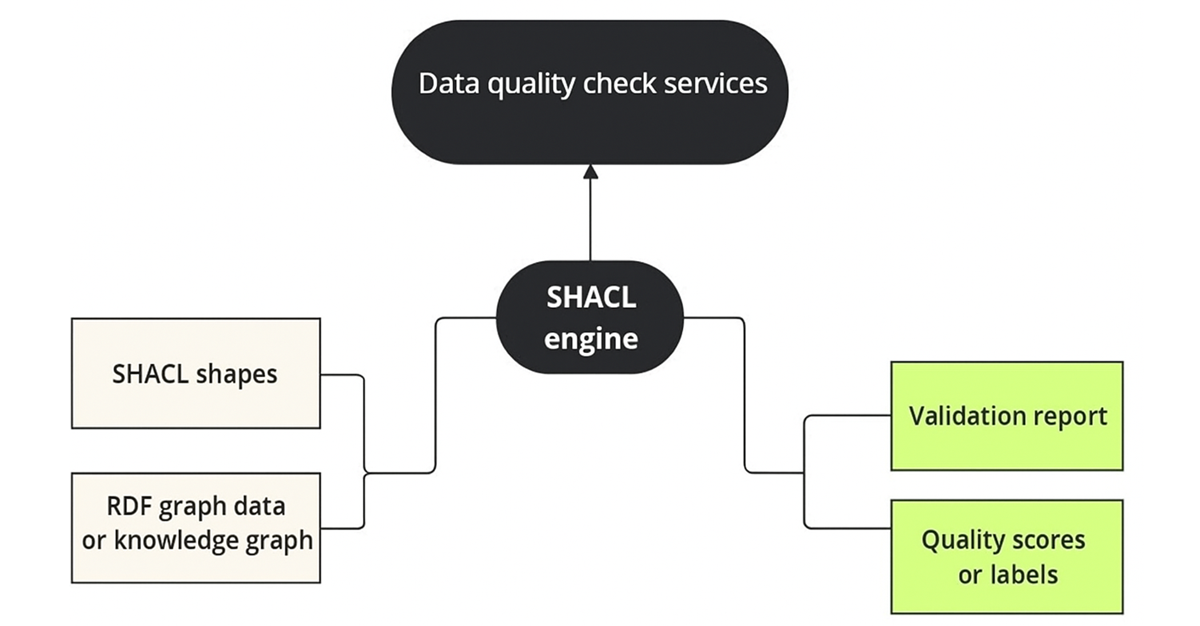
SHACL-based data quality check pipeline. RDF: Resource Description Framework; SHACL: Shapes Constraint Language.

These SHACL shapes are applied within SKGs (intrasource consistency and completeness) and PHKGs (across data source consistency and completeness), ensuring semantic and structural standardization at each stage. Figure 2 illustrates this validation process, applied at both the SHKG and PHKG levels.

The SHACL engine processes health data represented as an RDF graph—a structured format used to build KGs—by applying predefined constraints (SHACL shapes). These rules define the expected structure and content of the data, including semantic relationships and required elements. As the engine executes these rules, it produces an SHACL validation report that highlights data quality violations, such as missing values, incorrect data types, or inconsistent relationships. This validation step is critical for identifying and addressing quality issues before integration into the PHKG.

## Methods

### Study Design

To evaluate the robustness and effectiveness of the AIDAVA framework in detecting and managing data quality issues, we conducted a controlled experiment using the MIMIC-III (Medical Information Mart for Intensive Care-III) dataset, a publicly available and well-structured critical care database. While MIMIC-III offers a rich and diverse set of clinical variables, it does not contain the types of data quality issues typically encountered in real-world health information systems. As such, this study introduced artificial noise to simulate common completeness and consistency problems found in heterogeneous clinical data. This approach allows us to systematically assess how well the AIDAVA framework, particularly its SHACL-based validation rules, can identify quality issues under different conditions and at multiple stages of data integration. The following subsections describe the dataset, noise injection methodology, and the process of KG construction and validation.

### Data Source and Data Preparation

We used the MIMIC-III dataset due to its structured format and breadth of clinical variables, offering an optimal foundation for simulating real-world integration scenarios. The dataset includes deidentified health records from over 58,000 admissions. For this study, hospital admissions lacking relevant diagnosis or procedure codes (as required for SHACL validation) were excluded, resulting in a final cohort of 13,607 admissions. A total of 4 tables were used: PATIENTS, ADMISSIONS, DIAGNOSES_ICD, and CPTEVENTS.

Data cleaning was performed using OpenRefine [40], with date fields standardized to ISO 8601 format (YYYY-MM-DDThh:mm:ss+zz:zz) and gender values mapped to SNOMED CT codes to support interoperability and nonbinary classifications. These steps ensured alignment with the AIDAVA reference ontology and interoperability across sources.

### Adding Noise to the Dataset

To simulate real-world data quality challenges [41-43], artificial noise was introduced into the dataset based on 2 parameters: noise level and completeness ratio. Noise level (∈ [!0,1]) defines the proportion of KG statements impacted by errors. The completeness ratio specifies the share of this noise that results in missing values (as opposed to logically inconsistent entries). For example, a noise level of 0.50 and a completeness ratio of 0.25 imply that 50% of selected statements are altered, with 25% of them made incomplete and the remainder made inconsistent. Noise was introduced across 2 categories: consistency noise, reflecting logical contradictions, and completeness noise simulating missing information. All injections were performed in a reproducible manner using a fixed randomization seed.

### Consistency Noise

**Figure 3:**
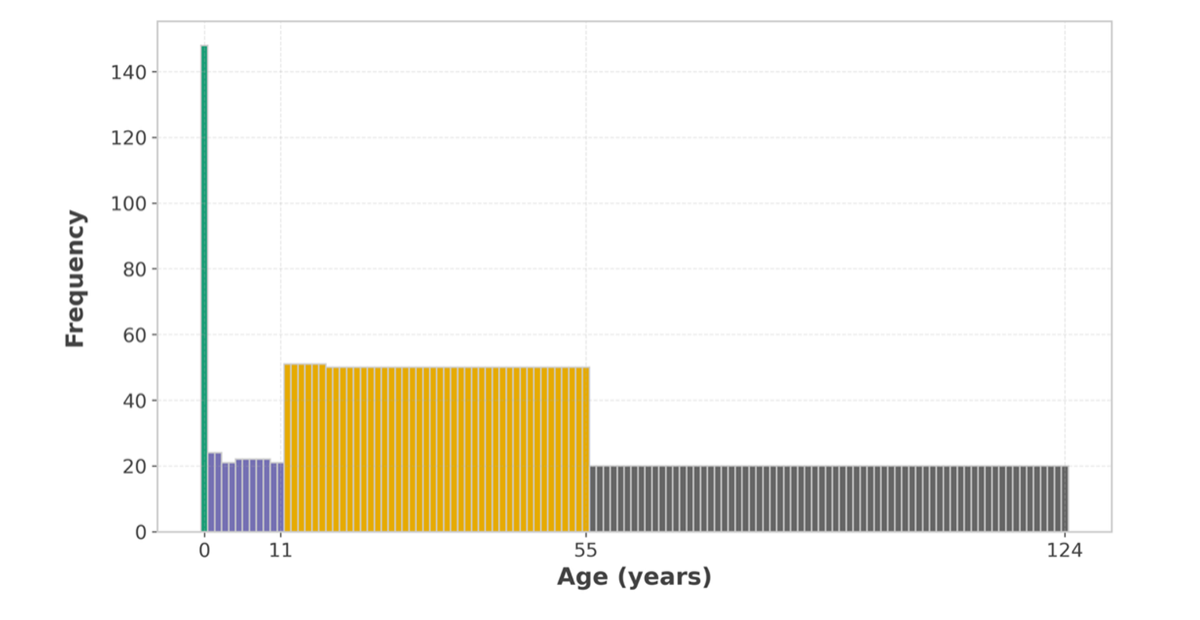
Age distribution histogram.

We targeted error types that frequently occur in clinical data entry or integration processes [41-43]. These methods included:

1. Gender swapping: male and female values in the PATIENTS table were randomly exchanged. This modification introduced inconsistencies in gender-specific diagnoses and procedures, affecting validation rules such as diagnosis for gender consistency and procedure for gender consistency. Gender values are sampled from a binomial distribution with 2 trials and success probability, *P*=.5, ensuring a balanced distribution of changes.
2. Age alteration: instead of randomly changing a patient’s age, we introduced interval-based errors by defining plausible age groups. These age groups were determined based on clustering patterns observed in diagnosis for age consistency and procedure for age consistency rules (Figure 3). In this implementation, age groups were defined as [!0,1] for infants, [!1,12] for children, [!12,56] for teens and adults, and [!56,124] for seniors. Birth years in the PATIENTS table were altered so that a new age was randomly selected from another age group, triggering violations in age-dependent diagnosis and procedure rules. To ensure realistic distribution, the selection of a new age group was sampled using a multinomial distribution with 4 trials, where the event probabilities were weighted based on the frequency of each age group in the dataset.
3. Swapping admission and discharge dates: in the ADMISSIONS table, admission, and discharge dates were swapped. This modification introduced inconsistencies relevant to time-sequence validation rules and triggered errors in age calculations that depended on the admission date.
4. Day-month swapping in dates: day and month values in date fields across PATIENTS, ADMISSIONS, and CPTEVENTS were randomly swapped, leading to invalid date formats where month values exceeded 12, or sequences where the chronological order of events became disrupted. These errors mirrored common data entry mistakes in hospital settings, where clinicians or administrative staff may mistakenly invert date components.
5. Inconsistencies in medical coding: we randomly changed diagnosis and procedure codes in the DIAGNOSES_ICD and CPTEVENTS tables. *ICD-9* (*International Classification of Diseases, Ninth Revision*) diagnosis codes and Current Procedural Terminology procedure codes were replaced with alternative codes randomly drawn from their respective rulesets. This change indirectly caused invalid gender and age violations in both diagnoses and procedures because certain codes are only applicable to specific demographic groups. To implement this, each affected record had its original code excluded and replaced with another randomly sampled value from the remaining choices, ensuring a uniform distribution of errors across the dataset.

### Completeness Noise

Missing data, based on real-world completeness issues, was introduced to replicate the effects of incomplete records on validation outcomes [41-43]. The affected tables and fields were:

1. Patient demographic records: missing values were introduced by randomly removing gender (GENDER) or date of birth fields in the PATIENTS table with equal probabilities of being chosen. This noise is intended to represent missing or deidentified patient information.
2. Hospital admission records: missing values were introduced by deleting admission or discharge timestamps (ADMITTIME, DISCHTIME) as well as admission or discharge locations (ADMISSION_LOCATION, DISCHARGE_LOCATION) from the ADMISSIONS table. As with demographic records, the field to be deleted is chosen with equal percentages of 25%. The selection of omitted records was performed randomly yet consistently across the experiment, preserving the dataset’s structural integrity while reflecting real-world gaps in clinical administrative case documentation.
3. Procedure records: missing values were introduced by removing procedure chart dates (CHARTDATE) or procedural codes (CPT_CD) in the CPTEVENTS table. Same with the others, equal probabilities of 0.5 decided which cell type should be deleted. The omission process was randomized across the experiment, replicating common record-keeping errors that lead to incomplete procedural documentation.
4. Diagnosis records: missing values were introduced by deleting *ICD-9* diagnosis codes (ICD9_CODE) from the DIAGNOSES_ICD table. As there is only 1 column type able to be deleted, it is chosen with the probability of 1, dissimilar to other categories of introducing completeness noise. This modification simulates errors observed by not typing *ICD* (*International Classification of Diseases*) codes for billing purposes.

While these noise injection scenarios may overlap in their effects, the incompleteness was introduced independently at this stage. The combined effects of the above, as they relate to triage and patient data acquisition progressions, are further elaborated and investigated in the following section. To account for the randomness in choosing the cell types to be deleted, this process was carried out in a reproducible manner using a fixed randomization seed.

### Data Integration and Data Quality Assessment

After noise injection, the dataset was mapped into SKGs using RDFCraft [44], aligning data elements to the AIDAVA reference ontology. These SKGs were then integrated into a unified PHKG, following a typical data ingestion workflow. We began with demographic information from the PATIENTS table, which provided key patient attributes. Next, temporal data from the ADMISSIONS table was incorporated, establishing admission and discharge events. This was followed by procedural details from the CPTEVENTS table, and finally, diagnostic information from the DIAGNOSES_ICD table. This stepwise integration reflects a typical hospital workflow, where patient registration occurs first, followed by admissions, treatments, and recorded diagnoses. At each step, SHACL validation rules were applied to assess data quality in terms of completeness and consistency. As each patient is modeled as an independent SKG, SHACL validation can be parallelized across patients, supporting scalable execution on large datasets. This staged validation aligns with levels 2 and 3 of the AIDAVA framework and reflects both intrasource and cross-source quality checks.

To evaluate the effect of the order of data addition on data quality, alternative sequences were also tested by changing the ingestion order (eg, loading procedures or diagnoses before demographics). This allows us to observe how quality issues propagate or get masked depending on integration order.

## Results

### Overview

The integration process followed a sequential order, beginning with the PATIENTS table, followed by ADMISSIONS, CPTEVENTS, and DIAGNOSES_ICD tables. The framework assessed completeness and consistency at each stage, tracking how data quality changed throughout the process. To evaluate the robustness of the framework, we also conducted an alternative integration sequence, starting with CPTEVENTS, followed by DIAGNOSES_ICD, ADMISSIONS, and finally PATIENTS.

In the following section, we first present the baseline data quality assessment without noise, establishing a reference for comparison. We then provide the final data quality scores after full integration across varying noise levels for both integration orders, highlighting key trends. A detailed breakdown of progressive changes at specific noise levels is available in Multimedia Appendix 1.

### Data Quality Assessment Without Noise

The baseline analysis, presented in Table 3, was conducted without artificial noise to establish a reference for data quality. The results showed that most dimensions and categories achieved nearly perfect quality scores, indicating that the integration process preserved data integrity and did not amplify errors. “Essential variable” completeness remained at 100% (13,607 of 13,607 admissions), confirming that all mandatory data elements were present in the KG. The domain consistency checks for diagnosis and gender, as well as diagnosis and age, yielded perfect quality scores. Similarly, data type adherence at all integration stages is achieved with no errors.

**Table 3:** Baseline data quality analysis results.

Dimension and category			PATIENTS		ADMISSIONS		CPTEVENTS		DIAGNOSES_ICD
**Completeness**									
	Essential variable (%)	100		100		100		100	
	Conditional variable (%)	N/A^a^		N/A		N/A		100	
**Consistency**									
	Time sequence (%)	100		99.9		99.9		99.9	
	Diagnosis for gender (%)	100		100		100		100	
	Procedure for gender (%)	100		100		100		100	
	Diagnosis for age (%)	100		100		100		100	
	Procedure for age (%)	100		100		99.89		99.89	
	Data type for property (%)	100		100		100		100	

^a^N/A: not applicable.

Although overall data quality is high, minor discrepancies were observed in specific categories. “Conditional variable” completeness category assesses whether all necessary concepts from different sources are properly integrated and complete. As SHACL rules evaluate conditional variables across every table, an incomplete graph would return 0% (0 of 13,607 admissions) quality score until the last stage of integration. To reflect this dependency, the “conditional variable” completeness scores for PATIENTS, ADMISSIONS, AND CPTEVENTS were marked as “N/A” because these values would not be meaningful to assess. Only at the DIAGNOSES_ICD stage could this category be properly evaluated, where all variable nodes will be reachable.

The procedure for gender and age categories received near-perfect scores of 99.99% (13,606 of 13,607 admissions) and 99.89% (13,593 of 13,607 admissions), respectively. These errors were not due to integration issues, but rather pre-existing errors in the MIMIC-III dataset. The errors in the “procedure for age” category originated from the dataset’s deidentification process, which assigned a few birth dates to years in the 1800s, resulting in implausible patient ages exceeding 300 years—a violation of age-related consistency rules. Similarly, errors in the “procedure for gender” category were traced to a data entry mistake in MIMIC-III, where a laparoscopic procedure on the oviduct or ovary (Current Procedural Terminology 58660) was attributed to a male patient. Additionally, time sequence consistency scored 99.9% (13,594 of 13,607 admissions), with errors primarily linked to discharge times recorded earlier than admission times. This discrepancy is a known artifact of the MIMIC-III data collection process, likely caused by inconsistent timestamp recording practices [45].

### Data Quality Assessment With Noise

#### Overall Trends Across All Noise Levels

**Figure 4:**
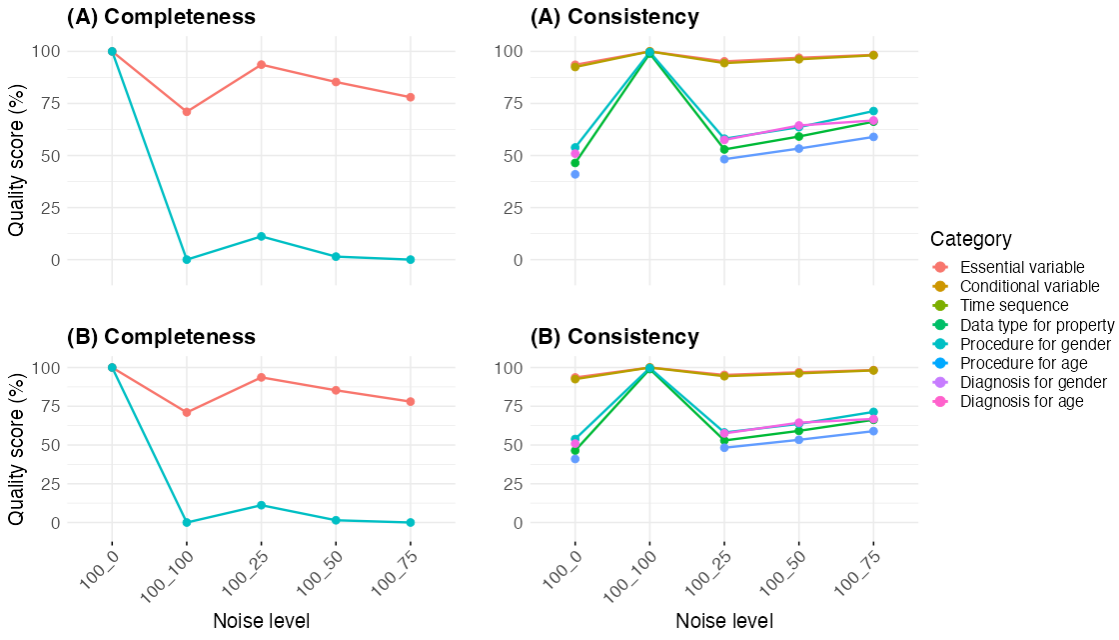
Final data quality scores after full integration.

To simulate real-world data quality challenges, we introduced artificial noise and repeated the integration process under 2 different orders. Figure 4 presents the final data quality scores after full integration for both orders. The results confirm that data quality scores remained consistent regardless of integration sequence, indicating that the order of data source integration does not alter overall data quality scores. While intermediate values may vary slightly, the progression of completeness and consistency follows the same overall patterns.

“Essential variable” completeness declined gradually from 100% to 71% (9661 of 13,607 admissions), with the largest drop occurring between 100_0 (100%) and 100_25 (93.65%), continuing to decrease at higher noise levels. “Conditional variable” completeness dropped sharply from 100% to 11.17% (1521 of 13,607 admissions) at 100_25, reaching near zero at higher noise levels, reflecting its strong dependence on cross-source relationships.

Time sequence and data type consistency improved throughout the integration, rising from 93.57% (12,732 of 13,607 admissions) and 92.51% (12,588 of 13,607 admissions) at 100_0 to 100% at 100_100. Procedure consistency for gender and age increased, starting at 46.43% (6136 of 13,607 admissions) and 53.86% (7329 of 13,607 admissions), respectively, and reaching 99.01% (13,472 of 13,607 admissions) and 99.68% (13,563 of 13,607 admissions) at 100_100. Diagnosis consistency for gender and age followed a different pattern, increasing steadily from 40.98% (5576 of 13,607 admissions) and 50.91% (6927 of 13,607 admissions) at 100_0 to 58.94% (8021 of 13,607 admissions) and 66.82% (9089 of 13,607 admissions) at 100_75, but both became unmeasurable at 100_100.

#### Detailed Analysis at 100% Consistency and 50% Completeness Noise (100_50)

**Figure 5:**
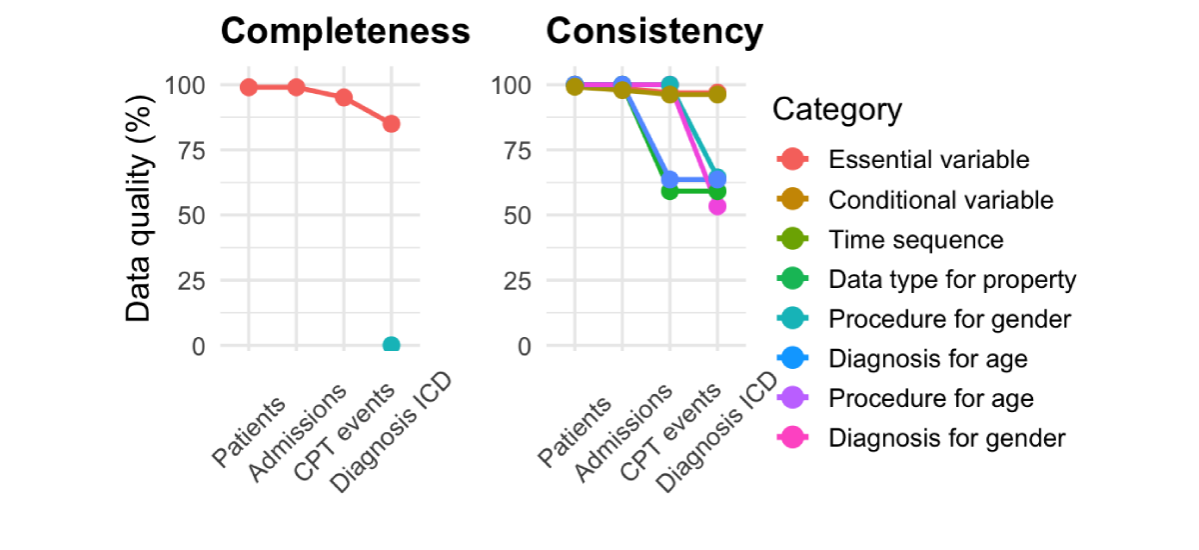
Data quality scores at each step of the integration process. CPT: Current Procedural Terminology; ICD: International Classification of Diseases.

To gain deeper insights into how data quality changes during data integration under specific noise levels, this section provides a detailed analysis of the 100_50 noise level. Figure 5 presents the data quality scores at each step of the integration process, illustrating how completeness and consistency evolve as new data sources are incorporated.

At this noise level, where consistency noise is set to 100% and completeness noise to 50%, all relevant KG statements (100%) are modified, with 50% of them containing missing data. The introduction of ADMISSIONS caused time sequence consistency to decrease slightly to 98.64% (13,422 of 13,607 admissions). With CPTEVENTS added, this metric dropped to 96.94% (13,191 of 13,607 admissions), where it remained stable after DIAGNOSES_ICD. Procedure consistency for gender declined to 59.14% (8047 of 13,607 admissions), while procedure consistency for age dropped to 63.63% (8658 of 13,607 admissions).

As expected, missing data impacted completeness measures. “Essential variable” completeness declined sharply, reaching 85% after DIAGNOSES_ICD. “Conditional variable” completeness remained “N/A” for PATIENTS, ADMISSIONS, and CPTEVENTS. Once DIAGNOSES_ICD was incorporated, “conditional variable” completeness increased slightly to 1.45% (195 of 13,607 admissions), reflecting a minimal recovery of required data.

An analysis of data quality trends across other noise levels is provided in Multimedia Appendix 1, detailing variations in completeness and consistency scores under different conditions.

## Discussion

### Principal Findings

Our findings demonstrate consistent patterns in data quality dynamics across all noise levels, characterized by a progressive degradation in consistency scores and a steady decline in completeness as noise levels increase. The category of “essential variable” completeness deteriorates gradually as noise accumulates, while “conditional variable” completeness declines more sharply due to its dependence on relationships across data sources. Meanwhile, “time sequence” and “data type consistency” remain relatively stable, whereas procedure and diagnosis consistency degrade significantly based on integration order, underscoring the importance of cross-source attribute alignment.

These trends confirm the adaptability of the AIDAVA data quality framework, which maintains stability in core structural checks (eg, time and data type validation) even as data volume and disorder increase. AIDAVA’s patient-level modularity enables SHACL validation to be performed independently per patient, supporting parallel processing and scalability. This design allows for incremental integration of new patient data without reprocessing the full dataset, making the framework suitable for real-time or batch-based deployments. However, the results also revealed that procedure and diagnosis consistency were more sensitive to integration order, emphasizing the need for targeted, domain-specific validation strategies during data merging.

To more explicitly frame these insights, the AIDAVA framework not only detects data quality issues with high granularity but also enables their continuous assessment throughout the integration pipeline. By embedding SHACL-based rules directly into the SKG and PHKG construction process, AIDAVA captures completeness and consistency violations both within and across data sources. This staged, rule-based validation empowers the framework to not only detect anomalies but also manage them by pinpointing their origin and timing—enabling early intervention and downstream reliability.

### Completeness and Conditional Dependencies in Data Integration

When examining the completeness dimension, the results reveal distinct patterns of degradation across our 2 categories: “essential variable” and “conditional variable” completeness. As expected, both categories are increasingly affected as the completeness ratio level rises. However, their rates of decline differ, highlighting important nuances in their behavior during the integration process. “Essential variable” completeness steadily declines, suggesting that core patient attributes are progressively impacted by noise—particularly those embedded in the PATIENTS table. In contrast, “conditional variable” completeness cannot be meaningfully assessed until the final integration step, as it depends on the availability of interconnected variables across all datasets. This explains why intermediate scores are marked as “N/A” and only become valuable after the final source (DIAGNOSES_ICD) is added.

These findings emphasize that “conditional variable” completeness is inherently linked to the integration process itself rather than noise alone. Unlike “essential variable” completeness, which is primarily affected by missing values within a single dataset, “conditional variable” completeness is more directly influenced by the presence or absence of cross-source relationships. As a result, its behavior differs from other completeness measures, demonstrating that missing values alone do not dictate “conditional variable” completeness trends—rather, it is the sequence and completeness of integrated sources that determine its final value.

### Consistency Challenges in Stepwise Data Integration

The consistency results reveal that step-by-step integration of data sources introduces challenges related to compliance with data type and time sequence consistency. However, these declines are relatively minor, suggesting that the overall format of the ontology remains stable and that temporal relationships are preserved throughout the integration process. Notably, time sequence consistency exhibits only minor fluctuations, reinforcing its resilience to integration steps. In contrast, the categories related to procedure consistency and diagnosis consistency show more pronounced declines, highlighting the critical role of integration order in determining data quality outcomes. The results indicate that integrating the CPTEVENTS table first has a substantial impact on procedure consistency, causing notable drops in gender and age consistency. Similarly, the integration of DIAGNOSES_ICD exacerbates diagnosis-related inconsistencies, suggesting that diagnostic data is particularly vulnerable to integration-induced errors. This trend highlights that procedure and diagnosis consistency are more reliant on cross-source relationships and attribute alignment than on other categories, such as data type and time sequence consistency.

The findings suggest that a case-specific data quality strategy is essential when integrating different types of health data. This underscores the need for tailored validation approaches that consider the vulnerabilities of various clinical data types during integration. This need for targeted strategies is evident both within and across data quality dimensions. For instance, within consistency, procedure, and diagnosis categories exhibit greater instability, highlighting that medical domain-specific content is more sensitive to inconsistencies than general data attributes such as timestamps or data types.

### Interdependencies Between Data Quality Dimensions

Beyond individual dimensions, these findings offer an in-depth perspective on the interrelationships between data quality dimensions, reinforcing the complex and dynamic nature of data quality in health data integration. While previous research has suggested that data quality dimensions are interrelated [42,46], our results offer a unique demonstration of how these interdependencies manifest in real-world integration scenarios. A key finding from the results is that consistency measures alone cannot be fully trusted without ensuring adequate completeness. The results reveal that missing data directly impacts the interpretability of consistency scores, making it difficult to determine whether the observed consistency is genuine or merely an artifact of incomplete data. When key information is missing, certain contradictions—such as conflicts between diagnoses and procedures—may go undetected, creating a false impression of data reliability.

These insights highlight the critical need for health data integration strategies that not only address individual data quality dimensions but also account for their interdependencies. Overlooking these relationships can lead to misleading assessments, where high consistency scores mask underlying data gaps, or missing values distort the true extent of consistencies. To ensure reliable data quality assessment, integration of data must prioritize completeness validation before consistency assessment, ensuring that inconsistencies are accurately detected rather than artificially hidden. By adopting an approach that considers both dimension-specific vulnerabilities and their cross-dimensional effects, we can enhance the integrity of downstream analyses, clinical decision-making, and secondary research applications—ultimately improving the reliability of integrated health data. Overall, our findings demonstrate that ontology-constrained SHACL validation enables interpretable, dynamic assessment of health data quality, with robustness across integration orders and degradation levels. This paper also extends prior research [47] (REF) by embedding semantic and clinical context into the data validation process. While existing tools such as OHDSI’s Data Quality Dashboard provide valuable population-level data quality checks after extract, transform, load into the OMOP common data model (eg, conformance, completeness, and plausibility), AIDAVA complements these approaches by offering patient-level validation throughout the integration pipeline. This facilitates early detection and localized resolution of issues that might otherwise remain hidden in aggregate-level analyses.

### Limitations

Several limitations must be acknowledged to contextualize the findings and guide future research directions. First, this study was conducted using the MIMIC-III dataset, a structured and deidentified critical care database. While this dataset provides a controlled environment for testing data integration, it does not fully capture the heterogeneity and complexity of live health care data environments. Relying on a single dataset also limits the generalizability of our findings, as results may differ across other institutions, coding practices, and patient populations. Although MIMIC-III was chosen for its structured format and public availability, access to additional well-curated benchmark datasets remains restricted due to privacy, licensing, and interoperability constraints. Future research should evaluate the framework on diverse, nondeidentified hospital datasets that better reflect real-world conditions. Second, this study simulated real-world data quality issues by introducing structured artificial noise, allowing for a systematic evaluation of the framework under different levels of data inconsistencies. However, artificial noise does not fully replicate the unpredictability of errors found in operational health data. Health datasets often contain context-dependent inconsistencies, undocumented missingness patterns, and human-introduced biases that cannot be easily simulated. Future research should explore how the framework performs when applied in a real-world scenario. Third, the AIDAVA framework evaluated consistency and completeness as the core data quality dimensions. However, other important dimensions, such as timeliness and uniqueness, were not explicitly assessed in this study. Future work should extend the framework to incorporate a broader range of quality dimensions, ensuring a more complete evaluation of integrated health data. Last, this study demonstrated the effectiveness of the AIDAVA framework in a controlled dataset; its scalability for large-scale, high-velocity health data integration was not examined in depth. Future research should investigate the framework’s performance in large-scale deployments. Finally, while AIDAVA was tested using structured simulations, adapting the framework for use with unstructured or semistructured real-world data (eg, electronic health records or clinical registries) remains a future challenge. These sources often include free text, heterogeneous coding, and loosely structured formats that require preprocessing steps (eg, terminology mapping). Investigating these adaptations is part of the ongoing research agenda within the AIDAVA project.

### Conclusions

This study evaluated the AIDAVA data quality framework for its effectiveness in detecting and managing data quality issues—specifically completeness and consistency—during the integration of heterogeneous health data. Using the MIMIC-III dataset, we simulated real-world challenges by introducing structured noise and systematically assessed how the framework performed across different integration sequences and noise levels. The staged SHACL-based validation enabled fine-grained analysis of data quality at multiple points along the integration pipeline. Our findings show that the AIDAVA framework successfully identifies both missing and inconsistent data elements and provides interpretable feedback at each stage of transformation. Together, these findings support the AIDAVA framework’s suitability for dynamic, life cycle–based data quality assessment. By enabling validation at each transformation step, the framework allows early detection, interpretable tracking, and strategic mitigation of data quality issues. Importantly, it encourages a holistic view of data quality—one that considers not only dimension-specific weaknesses but also how dimensions influence each other across the pipeline. As health systems increasingly rely on integrated datasets for clinical and research applications, frameworks such as AIDAVA provide essential infrastructure for building trust in secondary health data use.

### Future Research

Future work should focus on improving the AIDAVA data quality framework by aligning SHACL shapes with the OMOP common data model [41] and comparing multiple integration orders to assess their impact on data quality. Developing an OMOP-based RDF schema and implementing SHACL constraints would also support interoperability with OHDSI tools and promote the broader adoption of semantic, constraint-driven approaches to data quality. Expanding SHACL validation to laboratory results, prescriptions, and other hospital records will enhance its clinical applicability. Additionally, developing more realistic noise introduction methods will better simulate real-world inconsistencies, strengthening the framework’s robustness.

## Appendix

Multimedia Appendix 1 A detailed breakdown of progressive changes at specific noise levels and analysis of data quality trends across other noise levels.

## Abbreviations

AIDAVA: artificial intelligence–powered data curation and validation
FHIR: Fast Healthcare Interoperability Resources
ICD: International Classification of Diseases
ICD-9: International Classification of Diseases, Ninth Revision
KG: knowledge graph
LOINC: Logical Observation Identifiers Names and Codes
MIMIC-III: Medical Information Mart for Intensive Care-III
OHDSI: Observational Health Data Sciences and Informatics
OMOP: Observational Medical Outcomes Partnership
PHKG: personal health knowledge graph
RDF: Resource Description Framework
SHACL: Shapes Constraint Language
SKG: source knowledge graph
SNOMED: Systematized Nomenclature of Medicine
SNOMED CT: Systematized Nomenclature of Medicine–Clinical Terms
