# Supplementary material for: Assessing Data Quality in Heterogeneous Health Care Integration: Simulation Study of the AIDAVA Framework
Source: JMIR Med Inform. 2025 Nov 12;13:e75275. doi: 10.2196/75275 (PMC12779104; doi:10.2196/75275)
Supplement: Multimedia Appendix 1 [file medinform_v13i1e75275_app1.docx]

100% consistency and 0% completeness noise


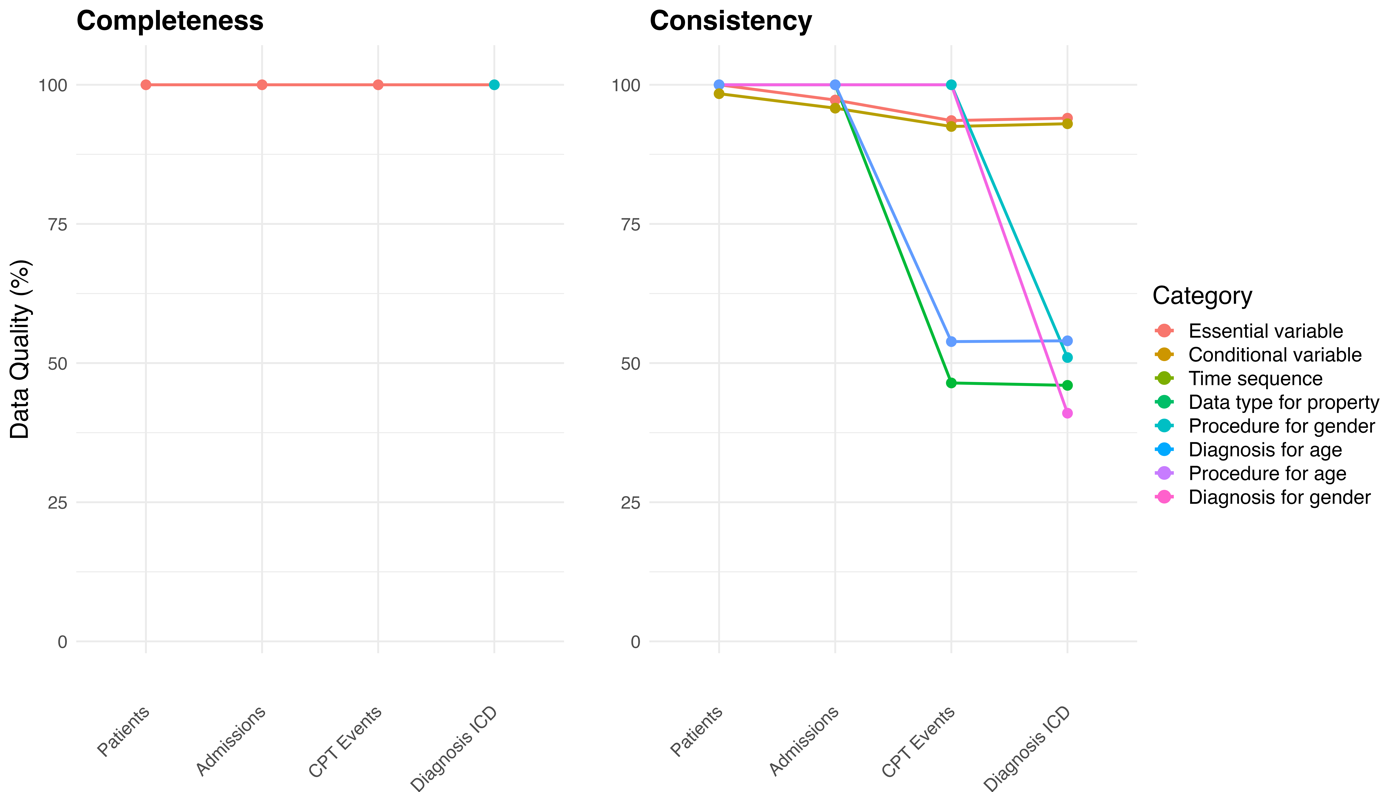


**Figure S1.** 100% consistency and 0% completeness.

As visualised in Figure S1, introducing 100% consistency and 0% completeness noise resulted in a progressive decline in consistency scores with each integrated table. However, completeness remained unchanged, as no data was missing. After integrating ADMISSIONS, time sequence consistency declined slightly to 97.26%. The addition of CPTEVENTS introduced inconsistencies in ‘procedure for gender’ and ‘procedure for age’, reducing their quality scores to 46.43% and 53.86%, respectively. These values remained stable following the integration of DIAGNOSES_ICD. However, this final step further impacted diagnostic consistency, with ‘diagnosis for gender’ dropping to 41% and ‘diagnosis for age’ consistency falling to 51%.

Figure 6: Data quality scores at 100% consistency noise and 0% completeness noise (100_0)

Interestingly, data type consistency declined to 92.51% after CPTEVENTS but showed a slight recovery to 93% after DIAGNOSES_ICD. This slight improvement in data type consistency after integrating DIAGNOSES_ICD suggests that some records may have aligned with expected formats at this stage.

100% consistency and 25% completeness noise


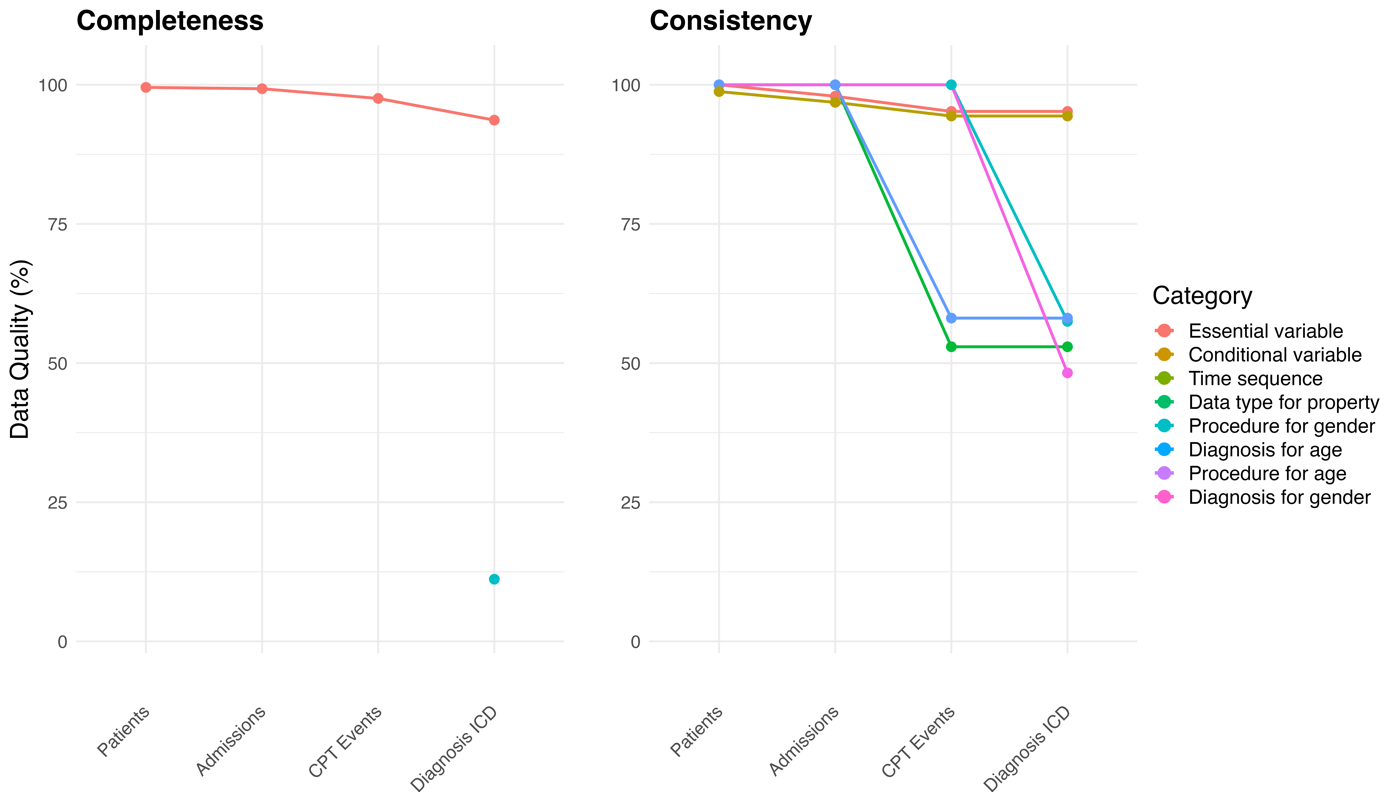


**Figure S2.** Data quality scores at 100% consistency noise and 25% completeness noise (100_25).

With 25% of the introduced noise consisting of missing, while 75% remained consistency errors, Figure S2 illustrates how the integration process was affected by both inconsistency and missing data. As ADMISSIONS was integrated, time sequence consistency declined slightly to 97.94%. When CPTEVENTS was introduced, procedure consistency for gender decreased to 52.93%, while procedure consistency for age declined to 58.05%. These values remained unchanged after integrating DIAGNOSES_ICD. The integration of DIAGNOSES_ICD further impacted diagnostic consistency, causing diagnosis-gender consistency to drop to 48.24% and diagnosis-age consistency to decline to 57.49%.

The presence of missing data had a visible impact on completeness. ‘Essential variable’ completeness declined at each step, reaching 93.65% after integrating DIAGNOSES_ICD. ‘Conditional variable’ completeness was marked as “NA” for PATIENTS, ADMISSIONS, and CPTEVENTS, as this dimension requires full dataset integration. However, once DIAGNOSES_ICD was integrated, ‘Conditional variable’ completeness reached 11.17%, indicating that some interrelated data elements became available in the final step.

100% consistency and 75% completeness noise


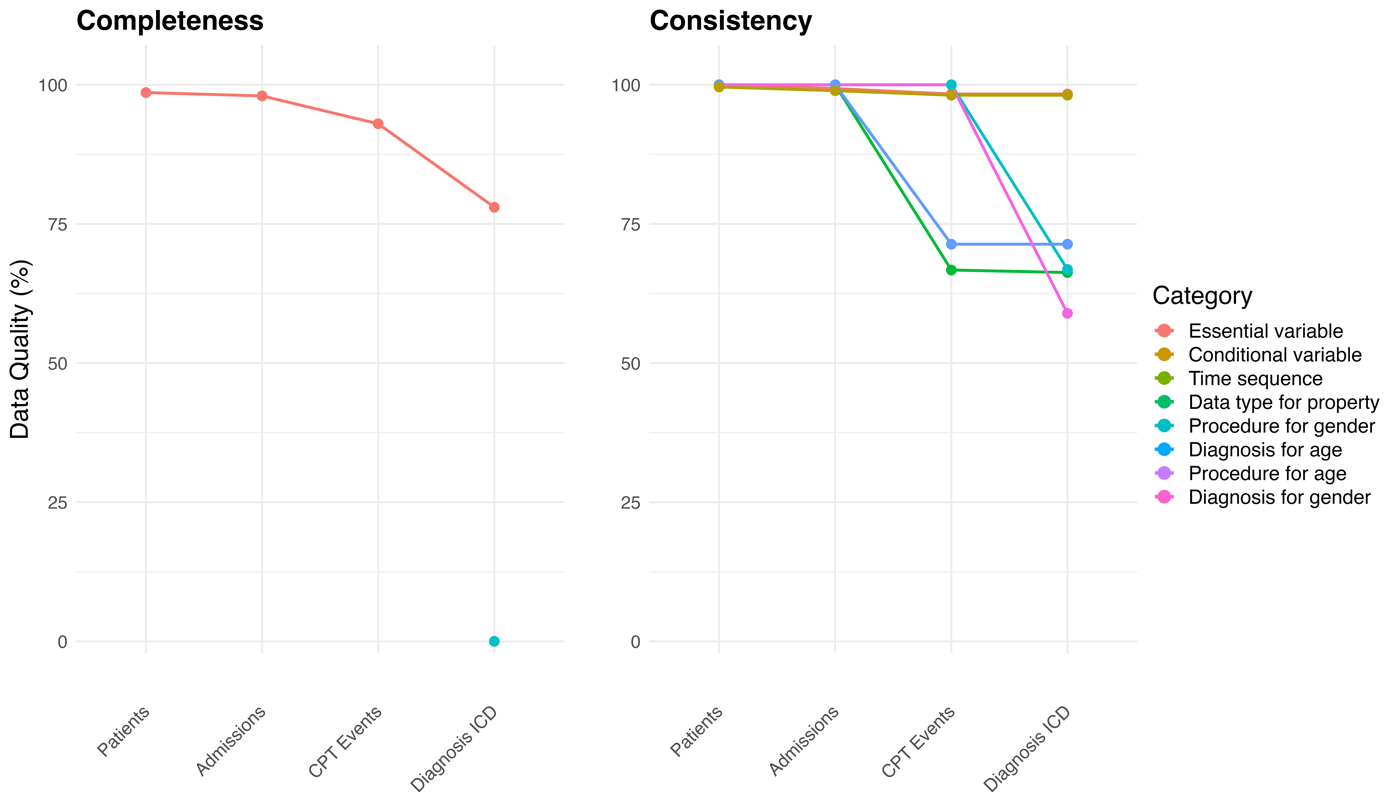


**Figure S3.** Data quality scores at 100% consistency noise and 75% completeness noise (100_75).

With 75% missing data, completeness degradation became the dominant effect (Figure S3). The integration of ADMISSIONS led to only a minor decline in time sequence consistency (99.32%), and this metric stabilized at 98.34% following CPTEVENTS. However, procedure consistency for gender dropped to 66.72%, and procedure consistency for age declined to 71.36%. ‘Essential variable’ completeness dropped to just 78% after DIAGNOSES_ICD, demonstrating the severe loss of data availability. After DIAGNOSES_ICD, ‘Conditional variable’ completeness remained close to 0%, showing that missing data severely impacted the availability of required interrelationships.

100% consistency and 100% completeness noise


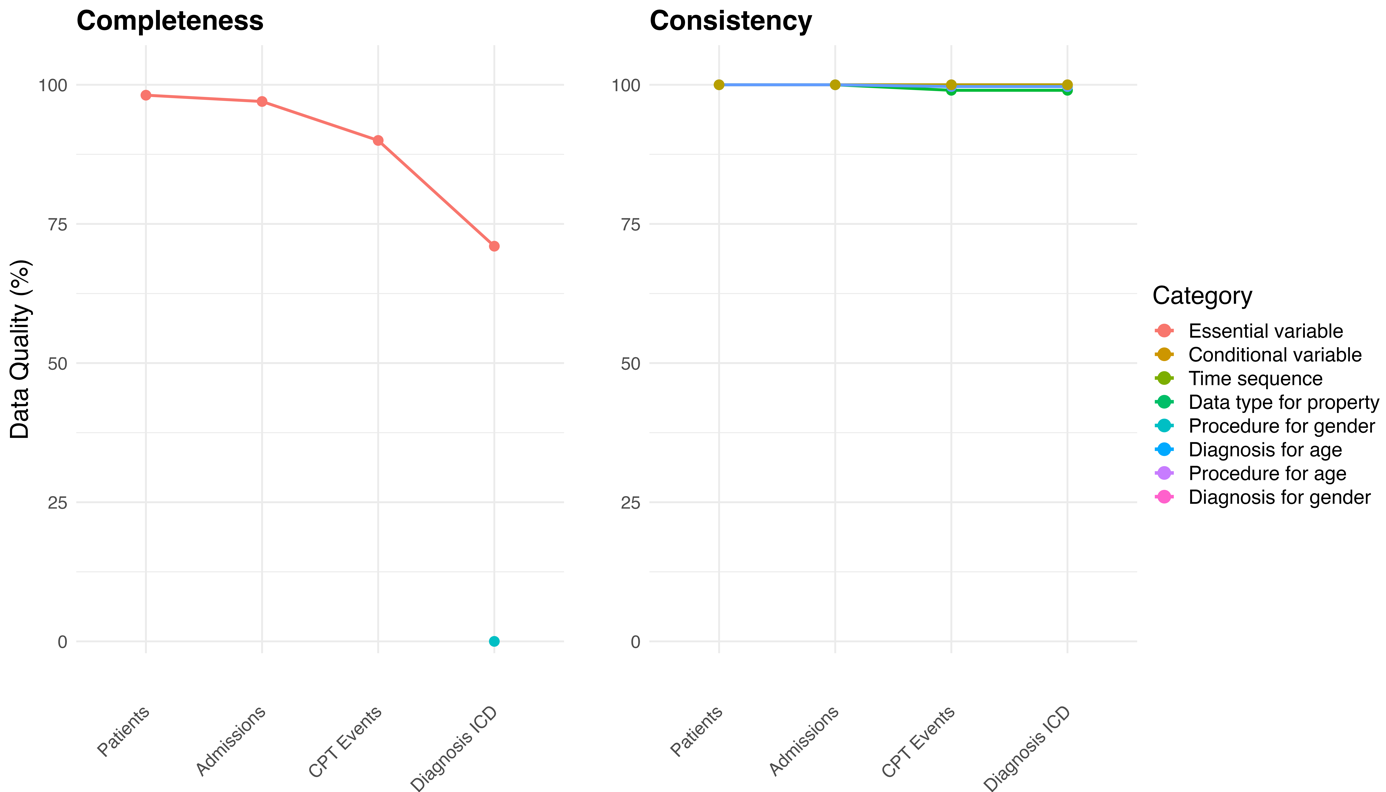


**Figure S4.** Data quality scores at 100% consistency noise and 100% completeness noise (100_100)

At 100% completeness noise, data loss was so severe that meaningful validation became impossible (Figure S4). Unlike previous noise levels, where inconsistencies could still be tracked, the overwhelming loss of data prevented effective validation. As CPTEVENTS was integrated, procedure consistency for gender and age could no longer be measured, as cross-source relationships had broken down. Time sequence consistency remained at 100% throughout, but this was misleading - the absence of data meant inconsistencies could not be detected rather than being resolved. Similarly, data type consistency remained at 100%, despite validation no longer being possible. The most severe impact was observed in ‘Essential variable’ completeness, which declined to 71% after DIAGNOSES_ICD - the lowest recorded across all noise levels. ‘Conditional variable’ completeness was entirely unassessable after integrating DIAGNOSES_ICD, as there was insufficient data to evaluate interdependencies between sources.
